# Guanabenz Treatment Accelerates Disease in a Mutant SOD1 Mouse Model of ALS

**DOI:** 10.1371/journal.pone.0135570

**Published:** 2015-08-19

**Authors:** Fernando G. Vieira, Qinggong Ping, Andy J. Moreno, Joshua D. Kidd, Kenneth Thompson, Bingbing Jiang, John M. Lincecum, Monica Z. Wang, Gerard S. De Zutter, Valerie R. Tassinari, Beth Levine, Theo Hatzipetros, Alan Gill, Steven Perrin

**Affiliations:** ALS Therapy Development Institute, Cambridge, Massachusetts, United States of America; University of Edinburgh, UNITED KINGDOM

## Abstract

Amyotrophic lateral sclerosis (ALS) is a progressive neurodegenerative disease characterized by loss of motor neurons. The mechanisms leading to motor neuron degeneration in ALS are unclear. However, there is evidence for involvement of endoplasmic reticulum (ER) stress and the unfolded protein response (UPR) in ALS, notably in mutant SOD1 mediated models of ALS. Stress induced phosphorylation of the eIF2 alpha subunit by eukaryotic translation initiation factor 2-alpha kinase 3 Perk activates the UPR. Guanabenz is a centrally acting alpha2 adrenergic receptor agonist shown to interact with a regulatory subunit of the protein phosphatase, Pp1/Gadd34, and selectively disrupt the dephosphorylation of the alpha subunit of eukaryotic initiation factor 2 (eif2alpha). Here we demonstrate that guanabenz is protective in fibroblasts expressing G93A mutant SOD1 when they are exposed to tunicamycin mediated ER stress. However, in contrast to other reports, guanabenz treatment accelerated ALS-like disease progression in a strain of mutant SOD1 transgenic ALS mice. This study highlights challenges of pharmacological interventions of cellular stress responses in whole animal models of ALS.

## Introduction

Amyotrophic lateral sclerosis (ALS) is a progressive neurodegenerative disease characterized by dysfunction of upper and lower motor neurons [1]. Until now, only riluzole has been approved as a treatment aimed at slowing ALS disease progression. It has been shown to extend patient life by 2–3 months. One approach to quickly address the unmet medical need presented by ALS is to “repurpose” therapeutics that are used to treat other conditions by testing them preclinically or clinically for ALS. Despite various successes with repurposed or repositioned drugs across myriad disease indications [2], results from this approach have thus far been disappointing with nearly a dozen failures in large multicenter randomized placebo controlled clinical trials for ALS between 2004 and 2014 [3]. Still, because repurposing remains an expeditious path toward development of therapeutics for people living with ALS, we have tested dozens of FDA approved therapeutics in the B6SJL(G93A-SOD1)/Gur1 model of ALS (hereafter referred to as G93A-SOD1 mice) to evaluate their potential as clinical candidates with limited success [4, 5, 6, 7].

Recently there have been reports of efficacy by guanabenz, an FDA approved antihypertensive drug, in G93A-SOD1 mice [8, 9]. Guanabenz is an alpha2 adrenergic receptor agonist shown to interact with a regulatory subunit of the protein phosphatase, Pp1/Gadd34, and selectively disrupt the dephosphorylation of the alpha subunit of eukaryotic initiation factor 2 [10].

Eukaryotic initiation factor 2 (eIF2) is essential for protein translation. Stress induced phosphorylation of the eIF2 alpha subunit by eukaryotic translation initiation factor 2-alpha kinase 3 Perk is one of three signal transduction pathways that activates the unfolded protein response (UPR) and reduces global protein production to levels more readily managed by available chaperone proteins for proper folding [11]. The other two arms of the UPR involve Atf6 and Ire1. UPR induction can enhance cellular survival [10]. However, while the presence of phosphorylated eIF2alpha reduces global translation and cellular demand for energy and enhances robustness against cellular stress, it also reprograms cellular transcription and translation machinery to ultimately increase levels of CHOP (aliases GADD153, Ddit3), a transcription factor that drives apoptosis [12]. This eIF2 mediated stress response circuit is a nexus point of a delicately balanced network optimizing cellular functionality, robustness, and controlled cell death in conditions of endoplasmic reticulum (ER) stress. Any pharmacological approach attempting to modulate cellular responses to stress caused by protein misfolding must balance these competing elements.

A series of genetic experiments have been performed with transgenic mice to study the role of the UPR in SOD1 mice. The first study crossed Perk haplo-insufficient mice with transgenic G85R-SOD1 mice. These Perk haplo-insufficient SOD1 mice displayed accelerated disease. This suggested that diminished capacity to respond to ER stress is deleterious [13]. Next they developed a SOD1 transgenic mouse harboring a Gadd34 mutation. The mutation retarded its capacity for dephosphorylation of the eIF2 alpha subunit. These mice had a delayed and less severe disease phenotype [14]. These results suggested that treatment with a drug that could also reduce GADD34 activity might be protective. Salubrinal, another small molecule Gadd34 inhibitor has been reported as efficacious in SOD1 mice [15]. However, it has a poor *in vivo* pharmacology profile. Guanabenz, a central acting drug with similar inhibitory effects on Pp1/Gadd34, was tested in SOD1 mice by the Roos lab and the Feng Lab (First Affiliated Hospital of Harbin Medical University in Harbin China) at doses of 8 mg/kg or 4 mg/kg every other day respectively. Both labs reported significant extension of survival. Additionally, guanabenz treatment of 4 mg/kg three times weekly demonstrated efficacy in a mouse model of prion disease [16].

The experiments and results detailed in this report were mostly completed before reports of efficacy by Jiang et al and Wang et al. and were catalyzed by the 2011 findings that guanabenz interferes with eIf2alpha dephosphorylation. Herein we report the effects of guanabenz on viability of SOD1 mouse fibroblasts exposed to tunicamycin induced cell stress. Further, we demonstrate guanabenz dependent effects on levels of UPR related transcripts and proteins from G93A-SOD1 spinal cord. Finally, we demonstrate disease acceleration by guanabenz in G93A-SOD1 mice by two treatment regimens: 1) 4.5 mg/kg/day by osmotic minipump continuous infusion and 2) a repeat of the 4 mg/kg *qod* protocol reported by the Feng Lab.

## Results

**Fig 1:**
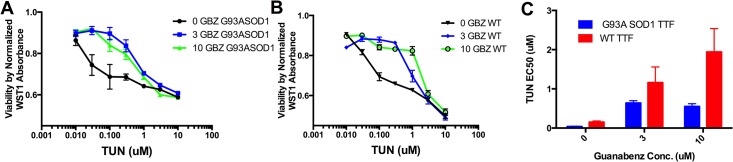
(A) WST1 absorbance as an indicator of cell viability in a tunicamycin challenge assay using fibroblasts derived from G93A-SOD1 mice. (B) WST absorbance indicator a tunicamycin challenge assay using fibroblasts derived from non-transgenic mice. (C) Tunicamycin EC50s as a function of guanabenz concentration in G93A-SOD1 and non-transgenic mouse fibroblasts.

Guanabenz treatment enhances viability of HeLa cells that have been stressed by tunicamycin [17]. Tunicamycin is a mixture of homologous nucleoside antibiotics that inhibits N-linked glycosylation and reproducibly induces the UPR in cells that are exposed to it [18, 19]. Here we repeated these experiments using primary fibroblasts derived from tail tips (TTF) from G93A-SOD1 mice and in TTF derived from littermates that harbor no mutant transgenes. These primary cells were used to take into account the role of SOD1 over-expression on the UPR induction and whether that would influence effects by guanabenz on the pathways. Similarly to the reported HeLa cell results, guanabenz treatment at low micromolar concentrations enhanced viability against tunicamycin induced cell death in fibroblasts in both WT TTFs and SOD1 TTFs at 3 and 10 uM concentrations (Fig 1A and 1B) and shifted the EC50 of tunicamycin significantly (Fig 1C). Guanabenz treatment shifted tunicamycin EC50 values higher to a greater extent in nontransgenic fibroblast experiments than in the G93A-SOD1 fibroblast experiments. The cell viability assay that was used relies on the cleavage of water soluble tetrazolium salt 1 (WST1) to formazan by mitochondrial dehydrogenase enzymes in viable cells. There is a measurable difference in absorbance when WST1 is converted to formazan and changes color. A greater shift to yellow indicates a higher number of viable cells.

**Fig 2:**
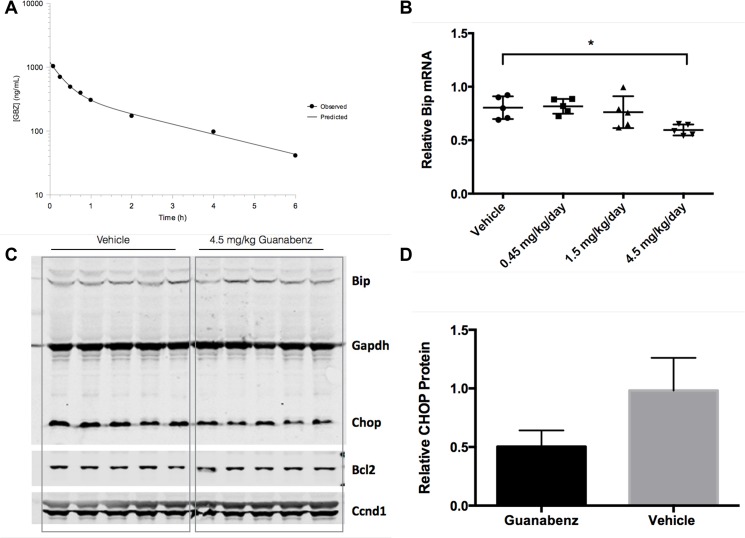
a) Guanabenz plasma drug levels in G93A-SOD1 mice after i.p. bolus injection of 10 mg/kg guanabenz. b) Relative mRNA expression in spinal cord of mice after continuous subcutaneous infusion of guanabenz at 0.45, 1.5, or 4.5 mg/kg or vehicle control. c) Western blot analysis of Bip, Chop, Bcl2, and Ccnd1 in spinal cord samples from mice treated with 4.5 mg/kg/day guanabenz or vehicle control. d) Relative quantitation of Chop protein determined by western blot in 4.5 mg/kg/day guanabenz treated mice or vehicle treated mice.

Next, *in vivo* experiments were completed to determine a well-tolerated treatment regimen that would maintain levels of guanabenz in spinal cord at low micromolar concentrations. A pharmacokinetics experiment was conducted in G93A-SOD1 mice where 10 mg/kg guanabenz was administered by intraperitoneal (i.p.) bolus injection. Plasma and spinal cord were harvested and analyzed. The plasma half-life of guanabenz was 1.8 hours (Fig 2A, Table 1). Peak spinal cord levels after a single 10 mg/kg bolus were approximately 7 uM. Using these pharmacokinetics parameters, a dosing regimen was modeled employing continuous infusion of approximately 4.5 mg/kg/day guanabenz by subcutaneous minipump that would achieve 1 uM continuous exposure of guanabenz in spinal cord.

**Table 1:** Guanabenz Mouse Pharmacokinetics. Guanabenz plasma pharmacokinetics parameters in G93A-SOD1 mice after i.p. injection of 10 mg/kg guanabenz.

Guanabenz PK Non-Compartmental Analysis		10 mg/kg
Parameter	Units	Plasma
Half-life Lambda z	hr	1.8
Tmax	hr	0.083
Cmax	ng/mL	1029.0

**Fig 3:**
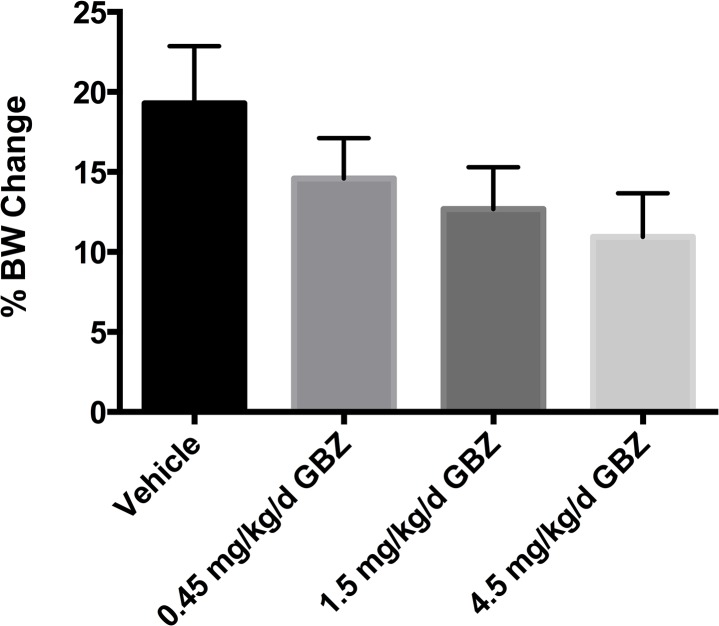
Average percent mouse body weight change after 28 days.

A study was then completed to determine if 28 days of guanabenz treatment at 0.45, 1.5, or 4.5 mg/kg administered by subcutaneous minipump would influence transcript levels of genes relating to the unfolded protein response in G93A-SOD1 mouse spinal cord. Mice were approximately 90 days old when tissues were harvested. Levels of Atf4, Bcl2, Bip, Ccnd1, and Chop transcripts were measured. Of these, only Bip was altered statistically significantly and only at 4.5 mg/kg/day. (Fig 2B, S1 Fig). Chop, Bcl2, and Bip protein levels in spinal cord of 4.5 mg/kg/day guanabenz-treated mice were compared to vehicle control by western blot. Only Chop levels were significantly altered, having been reduced by approximately 47% as measured by fluorescence intensity normalized to Gapdh fluorescence intensity (Fig 2C and 2D). In the same study, body weights were captured daily. Guanabenz appeared to reduce body weight gain in SOD1 mice at the dose levels being explored (Fig 3), though the differences were not significant by ordinary one-way ANOVA applying Dunnett’s multiple comparisons test. Further, the study observers noted that guanabenz-treated mice exhibited aggressive behavior during the first 14 days, but no toxicity was obvious during the 28 day study.

Guanabenz was then tested for efficacy against disease progression and survival endpoints in the G93A-SOD1 mice. 16 male and 16 female guanabenz-treated mice and 16 male and 16 female vehicle-treated mice were assigned. The cohorts were litter matched. Observers were blinded. The study was initiated when mice were approximately 50 days old. Osmotic minipumps were implanted delivering approximately 4.5 mg/kg/day in 6 ul of vehicle or 6 ul of vehicle alone. Of the 32 mice that were assigned to the guanabenz treatment cohort, only 20 survived until ALS related disease end-stage. The other 12 were euthanized according to IACUC protocols ensuring humane treatment of animals where 8 animals died during or did not recover from pump replacement surgery and 4 animals suffered injuries from self-mutilation (S1 Table). These animals were euthanized because they displayed signs of self-mutilation at the site of osmotic minipump implantation or because they did not recover from anesthesia after surgery. In comparison, the vehicle cohort included two non-ALS related death events where two mice did not recover from anesthesia following osmotic minipump replacement surgery. (S1 Table) An explanation for the increased mortality in the guanabenz treatment group is that both guanabenz and one component of our anesthesia cocktail, medetomidine, are alpha 2 agonists. The combination of the agents may not have been well tolerated in these mice.

**Fig 4:**
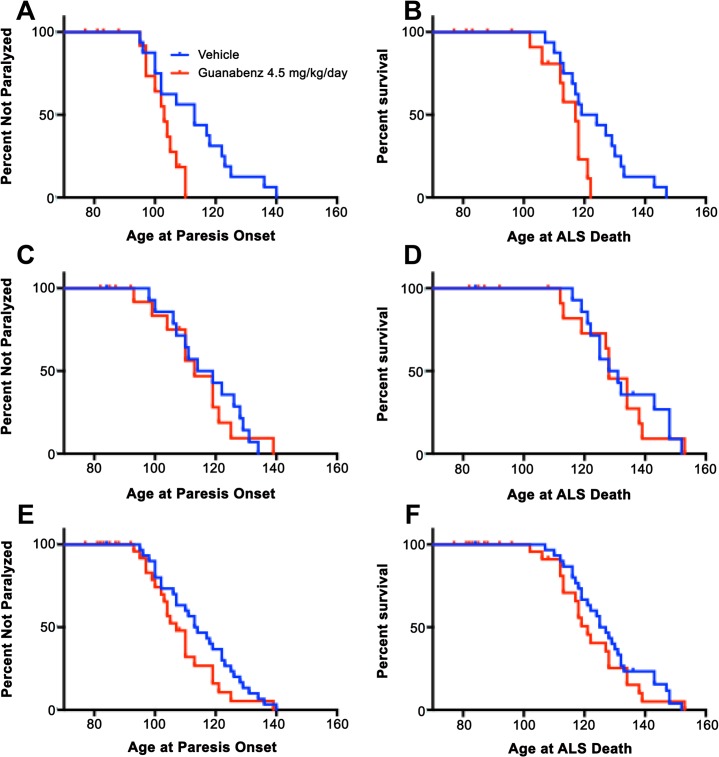
Kaplan meier curves of results from efficacy study 4.5 mg/kg/day subcutaneous infusion of guanabenz compared to vehicle control in G93A-SOD1 mice (A) onset of paralysis of male G93A-SOD1 mice, (B) survival of G93A-SOD1 mice, (C) onset of paralysis of female G93A-SOD1 mice, (D) survival of G93A-SOD1 mice, (E) onset of paralysis of all G93A-SOD1 mice, (F) survival of all G93A-SOD1 mice.

The remaining 20 guanabenz-treated and 30 vehicle-treated mice were analyzed for efficacy against ALS related endpoints. Guanabenz-treated male G93A-SOD1 mice exhibited accelerated age of onset of paresis and age at ALS-related death by 10 and 4.5 days respectively (p values = 0.02 and 0.02) (Fig 4A and 4B, Table 2, Table 3). No effect by guanabenz was observed on age at onset or age of death in female G93A-SOD1(Fig 4C and 4D, Table 2, Table 3).

**Table 2:** Guanabenz Efficacy Study Paralysis Onset Results Summary. Age at onset of paresis in 4.0 mg/kg qod efficacy study and 4.5 mg/kg/day efficacy study.

Study	Gender	Vehicle Treated Median Age at Paralysis Onset (days)	Median Age at Paralysis Onset (Guanabenz)	Trt—Veh (days)	P-value (Log-rank)
**4.5 mg/kg/day Guanabenz**	*Male*	*113*	*103*	*-10*	*0*.*0272*
**4.5 mg/kg/day Guanabenz**	Female	116.5	113	-3.5	0.7418
**4.5 mg/kg/day Guanabenz**	All	113.5	107	-6.5	0.1229
**4 mg/kg qod Guanabenz**	*Male*	*116*	*103*	*-13*	*0*.*0143*
**4 mg/kg qod Guanabenz**	Female	112.5	107	-5.5	0.051
**4 mg/kg qod Guanabenz**	*All*	*114*	*105*.*5*	*-8*.*5*	*0*.*001*

**Table 3:** Guanabenz Efficacy Study Survival Results Summary. Age of ALS related death in 4.0 mg/kg qod efficacy study and 4.5 mg/kg/day efficacy study.

Study	Gender	Vehicle Treated Median Death Age (days)	Guanabenz Treated Median Death Age (days)	Trt—Veh (days)	P-value (Log-rank)
**4.5 mg/kg/day Guanabenz**	*Male*	*121*.*5*	*117*	*-4*.*5*	*0*.*0266*
**4.5 mg/kg/day Guanabenz**	Female	129.5	128	-1.5	0.8278
**4.5 mg/kg/day Guanabenz**	All	126	121	-5	0.3808
**4 mg/kg qod Guanabenz**	*Male*	*128*	*116*	*-12*	*0*.*0149*
**4 mg/kg qod Guanabenz**	Female	122.5	118	-4.5	0.0976
**4 mg/kg qod Guanabenz**	*All*	*124*	*118*	*-6*	*0*.*0029*

After completion of the previously described studies, reports by other groups were published describing efficacy by guanabenz in G93A-SOD1 mice. In these reports, mice were treated on alternate days with either 4 or 8 mg/kg by i.p. administration. These reports compelled a follow up ALS disease endpoint efficacy study by our group. This was done to test whether pulsatile exposure might mitigate untoward effects observed in the continuous infusion study and reveal efficacy.

Our second guanabenz efficacy study aimed to repeat major attributes of the Jiang *et al* study with few alterations. B6SJL-G93A-SOD1 mice were used in both studies. Jiang *et al* only studied female mice (n = 30). Our experiment employed n = 32 female mice and n = 32 male mice. Jiang *et al* randomly assigned mice while our study employed a litter matched protocol because it has been shown that G93A-SOD1 mice from the same litter tend to live to approximately the same ages [6].

**Fig 5:**
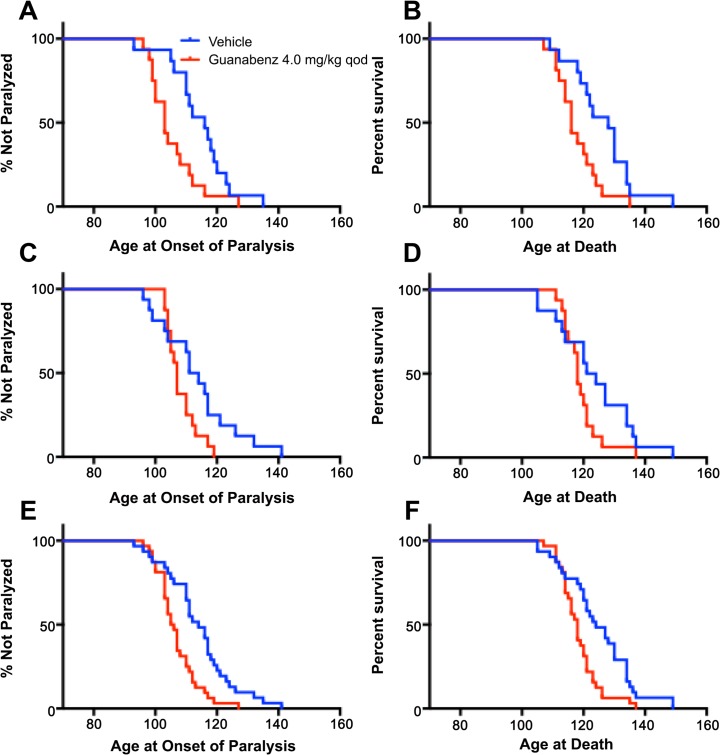
Kaplan meier curves of results from efficacy study 4.0 mg/kg qod intraperitoneal injection of guanabenz compared to vehicle control in G93A-SOD1 mice (A) onset of paralysis of male G93A-SOD1 mice, (B) survival of G93A-SOD1 mice, (C) onset of paralysis of female G93A-SOD1 mice, (D) survival of G93A-SOD1 mice, (E) onset of paralysis of all G93A-SOD1 mice, (F) survival of all G93A-SOD1 mice.

Unlike the osmotic minipump efficacy study, guanabenz-treated animals in the intraperitoneal injection *qod* study did not exhibit more aggressive behavior than vehicle-treated controls. All guanabenz-treated mice achieved ALS related death endpoints. Overall, guanabenz-treated mice exhibited earlier age at onset of paresis (8.5 days, p-value = 0.001) and earlier age at ALS-related death (6 days, p-value = 0.003) (Fig 5E and 5F, Table 2, Table 3). While both guanabenz-treated genders exhibited earlier median ages at onset and death, only males were statistically significant when analyzed alone with median onset 13 days earlier (p-value = 0.014) and median age at death 12 days earlier (p-value = 0.015). than the vehicle-treated cohort (Fig 5A and 5B, Table 2, Table 3).

## Discussion

ER stress and the UPR are involved in ALS. Numerous reports have identified activation of the main signaling branches of the UPR [20]. Enhanced phosphorylation of eIF2alpha has been demonstrated in post-mortem spinal cord of ALS patients. Also, XBP1, ATF4, and CHOP expression is increased in human ALS spinal cord [21, 22, 23].

This activation of the UPR is also modeled in transgenic mice over-expressing mutant human SOD1 genes which are known to cause heritable ALS [15, 24]. Genetic modulation of the UPR in transgenic mice over-expressing mutant human SOD1 significantly influences motor neuron disease symptom onset and lifespan. Mice over-expressing G85R mutant human SOD1 in the context of Perk haplo-insufficiency have earlier disease onset and reduced lifespans compared to G85R-SOD1 mice on a Perk+/+ background [12]. On the other hand, the same G85R-SOD1 mice crossed to mice with a mutated GADD34 gene on one allele have delayed onset and significantly prolonged survival [13].

One function of Perk is to phosphorylate the alpha subunit of eIF2 under stress conditions. This results in general translation suppression while enhancing transcription and translation of a number of cytoprotective genes and eventually, if stress is unabated, also genes and proteins that promote apoptosis. A later stage counterbalance to Perk and its phosphorylation of eIF2alpha within the UPR is Pp1/Gadd34, a phosphatase responsible for the dephosphorylation of eIF2alpha. The murine genetic crosses described above suggest that enhanced phosphorylation of eIF2alpha, its downstream effects on translation, and the selective expression of protective stress response proteins could result in improved disease outcomes in ALS animal models and perhaps in people living with ALS. This is in fact the outcome reported by two independent groups that tested the Pp1/Gadd34 inhibitor, guanabenz, in G93A-SOD1 mice [8, 9].

In direct contrast to these reports, we observed a statistically significant acceleration of onset of paresis and shortened lifespan of mice treated with guanabenz by two different treatment regimens. G93A-SOD1 male mice were more sensitive to the deleterious effects of guanabenz than their female counterparts.

The discrepancies may in part be explained by preclinical study design differences. For example, Jiang *et al* tested guanabenz only in female G93A-SOD1 mice while Wang *et al* do not report the gender breakdown of the mice in their studies. The most robust disease acceleration observed in our studies were in male mice. Also, while Jiang *et al* tested guanabenz in G93A-SOD1 mice on the B6/SJL mixed hybrid background as we did, Wang *et al* used the slower progressing C57B6 congenic strain. Finally, treatment start ages are different or incomparable across the studies. In our B6SJL background mice, we initiated treatment when mice were 50 days old while Jiang *et al* initiated treatment at 40 days of age and Wang *et al* initiated in C57B6 mice at 60 days of age. Finally, attempting to adhere to internationally established guidelines for testing of drugs in preclinical models of ALS, we litter-matched our cohorts to further reduce the risk of spurious results that can result from phenotypic outcomes clustering around litters [6, 25, 26]. The other groups did not mention this protocol detail.

There are a number of explanations why treatment with guanabenz might accelerate disease onset and shorten lifespan in G93A-SOD1 mice. First, by inhibiting dephosphorylation of eIF2alpha by Pp1/Gadd34, guanabenz treatment may have disrupted optimal intermittent activation of the UPR. The UPR is a tightly regulated program with built-in counterbalances. Initial stress induced phosphorylation of eIF2alpha by Perk and downstream impact on translation are later countermanded by Pp1/Gadd34 dephosphorylation of eIF2alpha. Without the appropriate balance, Perk mediated stress responses might ultimately accelerate apoptosis by way of Atf4/Chop signal transduction pathways. However, the G85R-SOD1/Gadd34 mutant cross mouse result suggests that Pp1/Gadd34 suppression can be protective. The inconsistency of our guanabenz pharmacological results and the genetic study results might suggest that UPR modulation at an earlier stage, perhaps as early as conception, might be essential for efficacy.

Another possible explanation for untoward effects observed in our guanabenz study might be its activities as an agonist of the alpha-2 adrenergic receptor. The dose levels used in our studies and of those in the other published reports are above those which cause hypotension in both hypertensive and normotensive rodent studies [27, 28]. Further, we observed aggressive behavior and reduced body weight gain in our guanabenz-treated mice indicating overall increased stress possibly independent of any UPR mechanism of action.

Because of the promising and informative results in genetic studies, further exploration of pharmacological agents which influence the UPR in *in vivo* models of ALS is warranted. Selective inhibitors of Perk have been developed [29]. Disease acceleration by Perk inhibitors might support the findings in SOD1/Perk haploinsufficient mice that Perk inhibition can accelerate disease. On the other hand, pharmacological Perk inhibition was protective in a TDP43 based drosophila model of neurodegeneration [30]. Another approach to better elucidate how to modulate the UPR in the context of ALS would be the testing of small molecule drugs that inhibit Pp1/Gadd34 activity but do not act as alpha2 adrenergic receptor agonists. While the small molecule, salubrinal, is a good molecular tool for cell-based work, it is suboptimal for whole animal pharmacology [10]. A molecule with similar or better activity against eIF2alpha dephosphorylation and with improved formulation, pharmacokinetics, and distribution attributes could be useful for research and possibly as a therapy in ALS or other neurodegenerative conditions.

Finally, it is important to consider that results from any modulation of the UPR, either pharmacological or genetic, in cellular or whole animal transgene over-expression models, may not fairly reflect the human disease condition caused by a single copy gene mutation or an undefined interplay of multiple genetic risk-factors and environment. Transgenic over-expression models, by their definition, apply an inordinate stress on cellular protein processing machinery that may skew results. This enhances the risk that preclinical results will not translate to those observed in humans.

## Materials and Methods

### Tail tip fibroblast isolation

A 1 to 2 cm length of tail-tips from 8-week-old wild-type and G93A-SOD1 mice were washed with 70% ethanol and PBS. The superficial dermis was peeled away and the remaining tissue was cut into 1 mm pieces using a scalpel. Five or six pieces were plated in one well of a six well plate and cultured with 2 mL of TTF medium (Dulbecco’s modified Eagle’s medium with 10% FBS and 50 units/mL penicillin/streptomycin) for 7–10 days. Cells migrating out were trypsinized and expanded into T25 flasks (passage 1).

### Tail Tip Fibroblast Tunicamycin Challenge Assays

TTFs were plated at 30–35% confluence in 96 well plates in 100 ul/ well using DMEM/high glucose and 10%fetal bovine serum using a trough and multichannel pipette. Cells were incubated overnight. Guanabenz in 50 ul low glucose DMEM and 1% FBS was added to cells in culture to achieve concentrations of 0, 3, or 10 ump in a final 200 uL of media. Cells were incubated for 1 hour. Tunicamycin in 50 ul of low glucose DMEM and 1% FBS was added to cells in culture to achieve concentrations of 0.01, 0.03, 0.1, 0.3, 1, 3, and 10 uM concentrations in 200 uL per well. Plates were then incubated for 48 hours @ 37°C and 5%CO_2_. 20 ul of WST1 substrate was then added to each well. Cells were incubated for 20 minutes. Absorbances were then read at 450nm.

### Animals

The studies were approved by the ALSTDI Institutional Animal Care and Uses Committee (IACUC) and in accordance with the Institute for Laboratory Animal Research (ILAR) Guide for Care and Use of Laboratory animals[31]. The SOD1-G93A mouse colony was derived from the B6SJLTgN(SOD1G93A)1Gur strain, obtained from The Jackson Laboratory (Bar Harbor, Maine) and originally produced by Gurney et al. The colony was currently being maintained by Biomedical Research Models, Inc. (Worcester, Massachusetts) by crossing hemizygous C57BL/6-SJL sires, harboring the transgene, with wild-type C57BL/6-SJL dams. Mice were shipped to ALS TDI at 35–45 days of age. Mice were allowed at least one week to acclimate to ALS TDI’s animal facility (12-h light/dark cycle at a temperature of 18–23°C and 40–60% humidity) before being assigned to a study. In all animal studies described herein, mice were singly housed. Food and water were provided ad libitum. The diet used was Teklad Global diet #2918 for rodents (Harlan Laboratories, Houston, TX). Drinking water was refreshed twice weekly.

### Mouse Genotyping

Genotyping was performed on ear tissue sampled from mice that were about 35 days old. 100μL of genomic DNA was extracted from approximately 15mg ear samples using the QIAmp Tissue DNA extraction protocol for the QIAcube HT automated liquid handler. gDNA quality and quantity was measured on a SpectraMax M5 plate reader taking readings at 260nm and 280nm. A relative quantification qPCR was used to probe for the human SOD1 gene, which is copy-number variable in the SOD1-G93A mouse model, using murine Gapdh as an endogenous control gene. A standard curve was created using genomic DNA from a mouse known to be a high-copy SOD1 mouse. This mouse was arbitrarily assigned 24 copies of the SOD1 gene, and for every qPCR the standard was run alongside the unknowns. The standard curves were fit with a two-phase decay regression in GraphPad Prism, and the unknown samples were assigned relative copy numbers by interpolation. Any mouse with less than 24 relative copies was not used. The SOD1 primer/probe set is custom-made from Life Technologies using the following sequences: GTAAATCAGCTGTTTTCTTTGTTCAGA for the forward primer, TTCACTGGTCCATTACTTTCCTTTAA for the reverse primer, and ACTCTCTCCAACTTTG for the VIC probe. The Gapdh primer/probe set is a Life Technologies Assay-On-Demand with Assay ID # Mm00186822_cn.

### Guanabenz Analytical Chemistry

Guanabenz acetate for all experiments described in this report was purchased from Sigma Aldrich (product# G110). For analytical chemistry standard preparations, five microliter aliquots were prepared in 1:1 water:acetonitrile and added to 45 uL of blank control plasma or blank spinal cord homogenate in a 96 deep-well plate. One hundred fifty microliters of acetonitrile containing 500 ng/mL of pyrimethamine as an internal standard and 0.1% formic acid were added to each well. The plate was vortexed vigorously and then centrifuged at 500x*g* for 30 minutes at 4 degrees C. One hundred microliters of the supernatant were pipetted into a new 96 well plate for LC-MS/MS analysis. Plasma and spinal cord homogenate samples from mice assigned to the guanabenz pk study were handled similarly without guanabenz spiking steps. Quantitation of guanabenz in tissues from the mice was interpolated by comparison of the signal to that generated by the standard curve. HPLC and mass spectrometer conditions for guanabenz were the same as those reported for detection of dexpramipexole in a previous report [32].

### Quantitative PCR

Tissue samples were homogenized in 1 mL Tri Reagent (Sigma #: T9424) by Precellys 24 homogenizer for two 20 second intervals. Next 0.1 mL of bromochloropropane was added the homogenate and mixed. After 10 minutes, samples were centrifuged at 12,000 x g for 15 minutes. RNA purification was carried out using Agencourt RNAdvanced kit on Biomek Laboratory Automation Workstation. RNA samples were than used to generate cDNA samples using Applied Biosystems High Capacity cDNA reverse transcription kits (Life Technologies #4368814). Quantitative PCR reactions were carried out using Applied Biosystems 7900HT Fast Real-Time PCR System. Target gene probe sets used were Atf4 (Life Technologies #Mm00515324_m1), Bcl2 (Life Technologies #Mm00477631), Bip (Life Technologies #Mm00516023), Ccnd1 (Life Technologies #Mm00432359), and Chop (Life Technologies #Mm00492097). Endogenous control probe sets used were Gapdh (Life Technologies # Mm99999915_g1), Canx (Mm00500330_m1), and Ubc (Life Technologies #Mm01201237_m1). Relative gene expression was determined using the GeNorm expression normalization algorithm [33]. Transformed relative expression data were then compared using GraphPad Prism software one-way analysis of variance and Tukey’s post-hoc test.

### Western Blots

Western blots were carried out using the following primary rabbit monoclonal antibodies from Cell Signaling, Inc: Bcl-2 rabbit mAb (#2870), BiP rabbit mAb (#3177), Chop rabbit mAb (#5554), Cyclin D1 (#2926). All of the above antibodies were incubated overnight at 1:1000 dilutions. A western blot was also carried out for detection of Gapdh using a mouse monoclonal antibody from Santa Cruz Biotechnology (#sc-47724).

Relative levels of Chop, Bcl2, and Bip proteins were compared by calculating fluorescent signal on western blots using the Leicor Odyssey Infrared Scanner. Chop, Bcl2, and Bip were all normalized with Gapdh as a loading control. Relative values of each target protein from treated mice were compared to vehicle control using t-test.

### Mouse Survival Efficacy Testing

We used general survival efficacy testing methods previously described. (Scott et al 2008) for the current studies. For both efficacy studies, transgene copy number was verified and mice were assigned to either drug treatment or vehicle treatment groups at 50 days of age. Groups were balanced with respect to gender (16 males, 16 females per group) and body weight within gender (mean weights at study start were typically within 0.3 grams for either gender between groups). Litters were evenly represented across treatment and vehicle groups. Observers were blinded to treatment groups.

For the intraperitoneal dosing study, vehicle was a 5% glucose solution prepared by diluting a 45% glucose stock solution (Mediatech, #25-037-CL, Lot #25037008) with water. For the treated cohort, 5.06 mg of guanabenz acetate salt (Sigma #G110, Lot #SLB2777V) was diluted into 10 mL of 5% glucose solution for a 0.4 mg/ml guanabenz free base solution on each day of treatment administration. Intraperitoneal injection volumes for both vehicle and guanabenz treated cohorts were 10 mL/kg body weight.

For the subcutaneous minipump implantation survival efficacy study, 191 mg of guanabenz acetate (Sigma #G110, Lot 081M1288V) was dissolved into 1.2 mL of 100% ethanol. Then, 3.6 mL of propylene glycol was added to the solution and vortexed. Last, 3.2 mL of water was added to the solution and vortexed. Osmotic minipumps (Alzet model 2004, Lot #10284–12) were loaded with the final solution.

Subcutaneous pumps were implanted when mice were approximately 50 days old. Exhausted pumps were removed and replaced every 28 days unless the mice exhibited a neuroscore of 2 in either hind-limb when the surgical procedure would have been carried out. For each surgical procedure, mice were treated with 0.1 mg/kg buprenorphine (Patterson Logistic Services: #07 850–2280) by i.p. injection. Mice were anesthetized by i.p. injection of ketamine (Patterson Logistic Services: #07 803–6637) and medetomidine (Patterson Logistic Services: #07 867–7105) cocktail including 1 mg/kg of each. Artificial tears ointment was applied to both eyes of each mouse. Depth of anesthesia was ascertained as sufficient when there was no response to pedal pressure. The dorsum of each mouse was shaved and incision sites sterilized. Skin incisions of 0.25 inches length were made between the scapulae. Subcutaneous pockets were made using sterile curved hemostats. Osmotic minipumps were wiped with 70% ethanol, allowed to dry, and then inserted into the subcutaneous pockets. Incisions were closed with 4–0 silk suture by simple interrupted pattern. Antibiotic ointment was applied to the closed incision sites. Mice were administered 1.5 mg/kg atipamezole (Patterson Logistic Services: 07 867–7097) by i.p. injection to reverse the sedative properties of meditomidine. The day following surgery, mice were treated with 0.1 mg/kg buprenorphine.

Mice were monitored for neurological disease progression according to the protocol previously reported (lithium paper). Neurological scoring procedures and body weight measurements were completed on a bench-top in the animal holding room. All neurological score and body weight data were captured by the custom ALSTDI Laboratory Information Management System (LIMS). End-stage mice were euthanized in a separate procedure room. Euthanasia for animals in all studies was carried out by CO2 chamber using 100% CO2 at a flow rate of approximately 20% of the chamber volume per minute. For the survival efficacy study, animals were euthanized by CO2 when they reach ALS related end-stage. This was defined by an inability of the mouse to right itself within 10 seconds when placed on its side. The observing technician is required to test the animal by placing the animal of both sides. Failure to right itself from either side results in the decision to euthanize. Mice were observed twice daily. Neurological scoring tests, including humane endpoint tests, were completed once daily. Kaplan-Meier curves and log-rank tests were used to analyze age at onset of paresis and survival data using Graphpad Prism6.

## Supporting Information

S1 Fig Additional Relative Abundances of Ccnd1, Chop, Atf4, and Bcl2 transcripts. Relative abundances of mRNAs of Ccnd1, Chop, Atf4, and Bcl2 in SOD1 mice treated for 30 days with vehicle control, 0.45, 1.5, or 4.5 mg/kg/day guanabenz.
(PDF)

S1 Table All raw neurological score data for Guanabenz Survival Efficacy Studies. (XLSX)

## References

[1] Rowland LP (1998) Diagnosis of amyotrophic lateral sclerosis. Journal of Neurological Science 160: Suppl 1:S6–24 10.1016/s0022-510x(98)00193-2 9851643

[2] Ashburn TT, Thor KB. (2004) Drug Repositioning: Identifying and Developing New Uses for Existing Drugs. Nature Reviews 3:673–683. 15286734 10.1038/nrd1468

[3] Mitsumoto H, Brooks BR, Silani V. (2014) Clinical trials in amyotrophic lateral sclerosis: shy so many negative trials and how can trials be improved? The Lancet Neurology 13: 1127–1138 10.1016/S1474-4422(14)70129-2 25316019

[4] Gurney ME, Pu H, Chiu AY, Dal Canto MC, Polchow CY, Alexander DD, et al (1994) Motor Neuron Degeneration in Mice That Express a Human Cu,Zn Superoxide Dismutase Mutation. Science 264: 1772–1774 8209258 10.1126/science.8209258

[5] Perrin S. Make mouse studies work. (2014) Nature 507: 423–425 24678540 10.1038/507423a

[6] Scott S, Kranz JE, Cole J, Lincecum JM, Thompson K, Kelly N, et al (2008) Design, power, and interpretation of studies in the standard murine model of ALS. Amyotrophic Lateral Sclerosis 9: 4–15 10.1080/17482960701856300 18273714

[7] Gill A, Kidd J, Vieira F, Thompson K, Perrin S. (2009) No Benefit from Chronic Lithium Dosing in a Sibling Matched, Gender Balanced, Investigator-Blinded Tiral Using a Standard Model of Familial ALS. PLoS ONE 4: e6489 10.1371/journal.pone.0006489 19649300 PMC2714460

[8] Wang L, Popko B, Tixier E, Roos RP. (2014) Guanabenz, which enhances the unfolded protein response, ameliorates mutant SOD1 induced amyotrophic lateral sclerosis. Neurobiology of Disease 71: 317–24 10.1016/j.nbd.2014.08.010 25134731 PMC4179984

[9] Jiang HQ, Ren M, Jiang HZ, Wang J, Zhang J, Yin X, et al (2014) Guanabenz delays the onset of disease symptoms, extends lifespan, improves motor performance, and attenuates motor neuron loss in the SOD1 G93A mouse model of amyotrophic lateral sclerosis. Neuroscience 277: 132–8 10.1016/j.neuroscience.2014.03.047 24699224

[10] Tsaytler P, Harding HP, Ron D, Bertolotti A. (2011) Selective inhibition of a regulatory subunit of protein phosphatase 1 restores proteostasis. Science 332: 91–4 10.1126/science.1201396 21385720

[11] Jiang HY, Wek SA, McGrath BC, Scheuner D, Kaufman RJ, Cavener DR, et al (2003) Phosphorylation of the alpha subunit of eukaryotic initiation factor 2 is required for activation of NF-kappaB in Response to Diverse Cellular Stresses. Molecular and Cellular Biology 23: 5651–5663 12897138 10.1128/MCB.23.16.5651-5663.2003 PMC166326

[12] Zinszner H, Kuroda M, Wang XZ, Batchvarova N, Lightfoot RT, Remotti H, et al (1998) CHOP is implicated in programmed cell death in response to impaired function of the endoplasmic reticulum. Genes and Development 12: 982–995 9531536 10.1101/gad.12.7.982 PMC316680

[13] Wang L, Popko B, Roos RP. (2011) The unfolded protein response in familial amyotrophic lateral sclerosis. Human Molecular Genetics 20:1008–15 10.1093/hmg/ddq546 21159797 PMC3033190

[14] Wang L, Popko B, Roos RP. (2014) An enhanced integrated stress response ameliorates mutant SOD1 induced ALS. Human Molecular Genetics 23: 2629–38 10.1093/hmg/ddt658 24368417 PMC3990163

[15] Saxena S, Cabuy E, Caroni P. (2009) A role for motorneuron subtype selective ER stress in disease manifestations of FALS mice. Nature Neuroscience 12: 627–36 10.1038/nn.2297 19330001

[16] Tribouillard-Tanvier D, Beringue V, Desban N, Gug F, Bach S, Voisset C, et al (2008) Antihypertensive Drug Guanabenz is Active In Vivo against both Yeast and Mammalian Prions. PLoS ONE 3: e1981 10.1371/journal.pone.0001981 18431471 PMC2291559

[17] Tsaytler P, Bertolotti A. (2013) Exploiting the selectivity of protein phosphatase 1 for pharmacological intervention. FEBS Journal 280: 766–70 10.1111/j.1742-4658.2012.08535.x 22340633

[18] Larsson O, Carlberg M, Zetterberg A. (1993) Selective killing induced by an inhibitor of N-linked glycosylation. Jornal of Cell Science 106: 299–307 10.1242/jcs.106.1.299 8270632

[19] Elbein AD. (1981) The tunicamycins–useful tools for studies on glycoproteins. TIBS 8: 219–21

[20] Matus S, Valenzuela V, Medinas DB, Hetz C. (2013) ER Dysfunction and Protein Folding Stress in ALS. International Journal of Cell Biology 2013: 674751 10.1155/2013/674751 24324498 PMC3845333

[21] Atkin JD, Farg MA, Walker AK, McLean C, Tomas D, et al (2008) Endoplasmic reticulum stress and induction of the unfolded protein response in human sporadic amyotrophic lateral sclerosis. Neurobiology of Disease 30: 400–7 10.1016/j.nbd.2008.02.009 18440237

[22] Ilieva EV, Ayala V, Jove M, Dalfo E, Cacbelos D, Povedano M, et al (2007) Oxidative and endoplasmic reticulum stress interplay in sporadic amyotrophic lateral sclerosis. Brain 2007: 3111–23 10.1093/brain/awm190 17716997

[23] Ito Y, Yamada M, Tanaka H, Aida K, Tsuruma K, Shimazawa M, et al (2009) Involvement of CHOP, an ER stress apoptotic mediator, in both human sporadic ALS and ALS model mice. 10.1016/j.nbd.2009.08.013 19733664

[24] Kikuchi H, Almer G, Yamashita S, Guegan C, Nagai M, Xu Z, et al (2006) Spinal Cord endoplasmic reticulum stress associated with microsomal accumulation of mutant superoxide dismutase-1 in an ALS model. PNAS 103: 6025–30 16595634 10.1073/pnas.0509227103 PMC1458691

[25] Ludolph AC, Bendotti C, Blaugrund E, Hengerer B, Loffler JP, Martin J, et al (2007) Guidelines for the preclinical in vivo evaluation of pharmacological active drugs for ALS/MND: report on the 142^nd^ ENMC international workshop. Amyotrophic Lateral Sclerosis 8:217–23 17653919 10.1080/17482960701292837

[26] Ludolph AC, Bendotti C, Blaugrund E, Chio A, Greensmith L, Loeffler JP, et al (2010) Guidelines for preclinical animal research in ALS/MND: A consensus meeting. Amyotrophic Lateral Sclerosis 11: 38–45 10.3109/17482960903545334 20184514

[27] Zhu H, Paul IA, Stec DE, Peeler DF, Piletz JE (2003) Non-adrenergic exploratory behavior induced by monoxidine at mildly hypotensive doses. Brain Research 964:9–20 12573508 10.1016/s0006-8993(02)03754-x

[28] Huang BS, Leenen FHH (1998) Both Brain Angiotensis II and “Ouabain” Contribute to Sympathoexcitation and Hypertension in Dahl S Rats on High Salt Intake. Hypertension 32: 1028–33 9856968 10.1161/01.hyp.32.6.1028

[29] Moreno JA, Halliday M, Molloy C, Radford H, Verity N, Axten JM, et al (2013) Oral treatment targeting the unfolded protein response prevents neurodegeneration and clinical disease in prion infected mice. Science Translational Medicine 206:206ra138 10.1126/scitranslmed.3006767 24107777

[30] Kim HJ, Raphael AR, LaDow ES, McGurk L, Weber RA, Trojanowski JQ, et al (2014) Therapeutic modulation of eIF2alpha phosphorylation rescues TDP-43 toxicity in amyotrophic lateral sclerosis disease models. Nature Genetics 46:152–60 10.1038/ng.2853 24336168 PMC3934366

[31] Committee for the Update of the Guide for the Care and Use of Laboratory Animals, Institute for Laboratory Animal Research (Ed.). (2010). Guide for the Care and Use of Laboratory Animals Eighth Edition. National Academies Press.

[32] Vieira FG, LaDow E, Moreno A, Kidd JD, Levine B, Thompson K, et al (2014). Dexpramipexole Is Ineffective in Two Models of ALS Related Neurodegeneration. PLoS ONE 9(12): e91608 10.1371/journal.pone.0091608 25526593 PMC4272269

[33] Vandesompele J, De Preter K, Poppe B, Van Roy N, De Paepe A, Speleman F. (2002) Accurate normalization of real-time quantitative RT-PCR data by geometric averaging of multiple internal control genes. Genome Biol. 3(7):RESEARCH0034 12184808 10.1186/gb-2002-3-7-research0034 PMC126239

