# Supplementary figures and images for: Guanabenz Treatment Accelerates Disease in a Mutant SOD1 Mouse Model of ALS
Source: PLoS One. 2015 Aug 19;10(8):e0135570. doi: 10.1371/journal.pone.0135570 (PMC4545826; doi:10.1371/journal.pone.0135570)

**Relative Ccnd1**

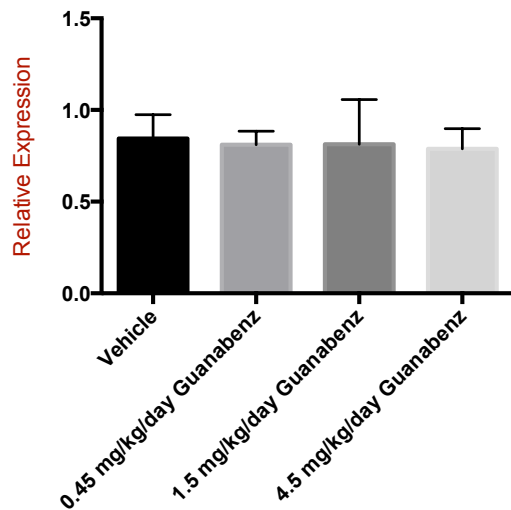

**Relative Chop**

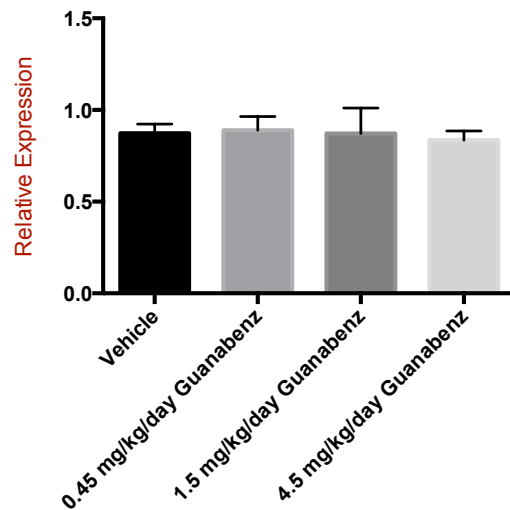

**Relative Atf4**

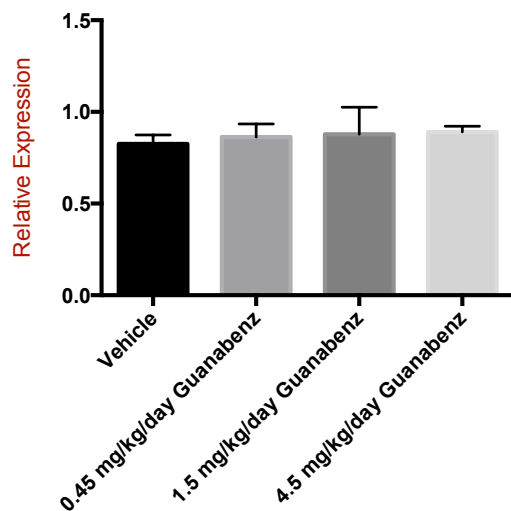

**Relative Bcl2**

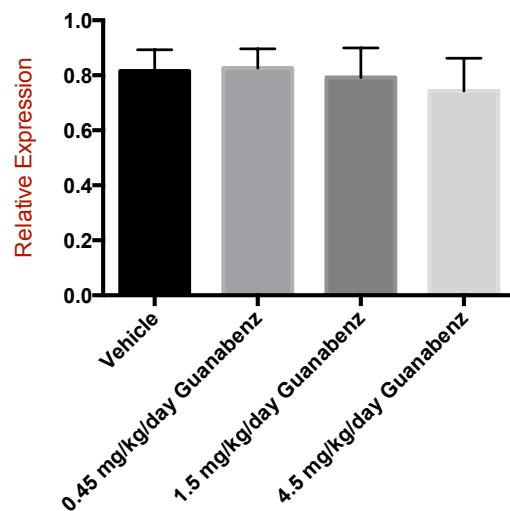

Supplement: S1 Fig — Relative abundances of mRNAs of Ccnd1, Chop, Atf4, and Bcl2 in SOD1 mice treated for 30 days with vehicle control, 0.45, 1.5, or 4.5 mg/kg/day guanabenz. (PDF) [file pone.0135570.s001.pdf]
